# Ankle Height Preservation with the Hind Foot Nail and Iliac Crest Bone Blocks in Patients with Sequelae of Partial or Complete Talus Bone Loss

**DOI:** 10.5704/MOJ.2111.014

**Published:** 2021-11

**Authors:** C Gunasekeran, K Bhowmick, B Ramasamy, TS Jepegnanam

**Affiliations:** 1 Department of Orthopaedics, Christian Medical College and Hospital Vellore, Vellore, India; 2 Department of Orthopaedics, Royal Adelaide Hospital, Adelaide, Australia

**Keywords:** hind foot arthrodesis, staged management, talar bone loss, infection

## Abstract

**Introduction:**

The management of talus bone loss in trauma is difficult and unsatisfactory. This study assessed whether the height of the ankle was preserved when entire or partial talar bone loss was managed with hind foot intramedullary nail augmented with autogenous rectangular or trapezoidal cortico-cancellous bone blocks from the iliac crest in the presence of active or latent infection.

**Materials and methods:**

Four patients were included in the study from January 2011 to December 2017. In the first stage, all four patients underwent debridement of the ankle, total or partial excision of the talus, and antibiotic-loaded bone cement spacer (ALBC) placement in the ankle joint. The second stage of the arthrodesis procedure was initiated six to eight weeks after the primary procedure, where these patients underwent arthrodesis with hindfoot nail and bone blocks from the iliac crest.

**Results:**

All patients were followed-up for an average of 17.6 months (range 12.0 – 32.0 months). The arthrodesis site had united in all these four patients. The AOFAS scores were satisfactory in all patients. One patient underwent nail removal after the arthrodesis site had united.

**Conclusions:**

The hind foot nail with iliac crest bone block maintains the ankle height and ensures successful arthrodesis. In patients with partial/ complete bone loss with suspicion or confirmation of infection, staging the arthrodesis procedure minimises the chance of complications.

## Introduction

The management of Type 3 and 4 talus fracture-dislocations with extrusion is difficult and unsatisfactory. These injuries are usually violent and are associated with severe soft tissue damage. The resultant combination of gross contamination, soft tissue damage, and attempts at restoring the normal ankle anatomy either by replacing the extruded talus in its original position or by fixation gives a poor outcome^1-3^. Treatment for these injuries is either retention/ fixation of the talus, preserving the ankle anatomy, or excision of the talus with arthrodesis of the tibio-calcaneal joint^2,4-8^. These contrasting methods of management of talus bone loss have found acceptance in orthopaedic practices worldwide^2,7,8^. Preservation of the talus maintains the ankle architecture and height but is complicated by high rates of infection, late-stage osteonecrosis, and multiple procedures^3,8^.

When there is a complete loss of the talus or restoring the original anatomy is impossible, tibio-calcaneal arthrodesis becomes the keystone of treatment, which can be achieved by internal as well as external fixation^9-11^. Screws, plates, and intramedullary nails are used for internal fixation and compression^12-15^. External fixation devices used for tibio-calcaneal arthrodesis are uniplanar or circumferential compression frames^10,16,17^. Internal fixation is found to be superior to external fixation as they are biomechanically stable and have higher union rates^11,18^. Union at the arthrodesis site is augmented by autogenous bone graft and allografts^19,20.^ Metallic or ceramic prosthetic replacement of the entire talus has been described with fair results^21-23^.

This aim of this study is to assess the outcome of management of the sequelae of talus bone loss (complete or partial) with hind foot nail device and autogenous rectangular/trapezoidal bone graft blocks from the iliac crest to reconstruct the height of the ankle joint in the presence of confirmed/suspicion of infection.

## Materials and Methods

This retrospective study was approved by our Institutional review board. Four non-consecutive patients were identified from the hospital database of a Level 1 trauma referral centre from the year January 2011 to December 2017 and included in this study. Informed consent was taken from all these patients. The inclusion criteria for this study were patients with partial/complete talectomy and its sequelae of infection, bone loss, and soft tissue loss requiring flap coverage. The exclusion criteria were patients with prior hind foot arthrodesis or who were unwilling to participate in this study. All were male with an average age of 28.2 years, and were involved in fall from height with a compound injury of the ankle. None of the patients had any co-morbidity.

Patient 1 was treated by us from the initial time of the injury, wherein, the entire extruded part of the talus was excised leaving a remnant of the talus with soft tissue attachment in the ankle joint. In Patient 2, the talus was completely excised and ALBC placed in the ankle joint before presenting to us. In patients 3 and 4, ORIF of the talus with soft tissue cover was performed at different hospitals. Radiographs of these two patients indicated a failure of fixation, devascularisation with features of chronic infection. All the patients except Patient 1 had undergone an average of two surgeries before undergoing staged arthrodesis at our institution. Pre-operative markers of infection were performed for all these patients.

In all the four patients, we staged the arthrodesis procedure. In the first stage, the ankle joint was debrided and the entire talus or the infected and de-vascularised part of the talus was excised. In Patient 2, the talus was already excised and filled with ALBC. In this patient, we debrided the ankle joint and re-applied the ALBC containing 2g of Vancomycin and Meropenem, respectively. In patients 1, 3 and 4, debridement of the ankle joint was performed and the de-vascularised, infected part of the talus was excised till bleeding healthy bone was achieved. The ankle joint with the remnant talus was filled with ALBC as mentioned above. The second stage of the arthrodesis procedure was initiated six to eight weeks after the primary procedure in which these patients underwent arthrodesis with hind foot nail and bone blocks from the iliac crest. In the post-operative period, the clinical and radiological follow-up of these patients was done from six weeks onwards of the arthrodesis procedure.

Bony union was defined radiologically as at least three cortices union in AP and lateral views during the follow-up period which was assessed by two of the authors at any time^24^. A functional assessment of the treatment was done by the AOFAS scores (maximum score of 86)^25^. The complications of infection, wound necrosis and, implant failure were noted.

The ankle joint was exposed by the anterolateral approach^26^. The skin incision was made 5cm proximal to the ankle joint and continued 5cm distally in line with the base of the 4th metatarsal. Previous posteromedial or posterolateral skin incisions over the area were ignored. If the previous skin incision was present on the anterior ankle region, it was included in the universal incision. Sinuses over the ankle region were curetted and debrided. The extensor tendons and neurovascular structures of the anterior compartment of the ankle were retracted medially and the joint capsule was incised in line with the skin incision, exposing the distal tibia, ankle joint up to the tibiofibular joint and the talonavicular joint. The ankle joint was debrided and depending on the osteonecrosis or osteomyelitis of the talus; it was partially or completely excised in cases with infection. Patient 2 had already undergone complete talectomy secondary to multiple previous surgeries performed at a different hospital, whereas, Patients 1, 3 and 4 underwent debridement of the talus till bleeding bone was achieved. The ankle joint was stabilised with joint spanning external fixator or below knee POP splints in all four patients. The void of the talar dome area was filled with Gentamycin cement [DePuy CMW^™^, USA] mixed with 2g each of Vancomycin and Meropenem each, respectively. This antibiotic-loaded bone cement (ALBC) was applied to control the infection and maintaining the space and architecture of the talar dome, to be filled with bone graft in the second stage of the surgery^27^. The wound was closed in layers with a suction drain. In all four patients, the debrided tissues and bone were sent for antimicrobial cultures.

In the post-operative period, patients were administered with culture appropriate intravenous or oral antibiotics for six weeks. These patients were allowed to walk on crutches without applying weight on the affected limb.

In the second stage, the ankle joint was exposed through the same incision as previously described and the cement spacer was removed. The tibial plafond and the talar remnant or calcaneum were curetted till bleeding cancellous bone was visualised. The gap between the distal tibia and the talus remnant or calcaneum (such as in Patient 2 where the talus was completely excised) was measured. The gap was 2.5cm, 3.5cm, 3cm, and 3.2cm in Patients 1, 2, 3, and 4, respectively. Two rectangular or trapezoidal bone blocks were harvested from each of the patient’s iliac crest with a standard width of 3cm and length according to the gap as measured. The width of these bone blocks was kept at a standardised 3cm to accommodate the width of the tibial plafond. These bone blocks were packed in the void of the tibiotalar joint lengthwise, maintaining the height of the ankle joint. Tibio-calcaneal arthrodesis was now performed using the hind foot nail, maintaining the bone blocks in position, so that one of the screws into the nail would pass through the bone blocks, holding them in position. The ankle was held in 0° - 5° of plantar flexion and 5° - 10° of valgus for final fixation in all the patients. In Patients 2 and 3, we used hind foot nails [TriGen, Smith and Nephew, USA], whereas, in Patients 1 and 4, we used retrograde femur nails [TriGen meta-nail, Smith and Nephew, USA] for fixation as shown in Table I.

**Table I:** Treatment details, functional score and sequelae

Serial Number	1st Stage Surgery	Infection	Culture	Antibiotics	Talar Lesion	2nd Stage Surgery (Arthrodesis)	BG	Complications	Follow-up months	AOFAS Score (Max= 86)	Sequalae
1	Debridement, Partial Excision Talus, Antibiotic Spacer, And Joint Spanning External Fixator	NIL	NIL	Cap cephalexin six weeks	Partial talus excision	Trigen retrograde femur nail	Iliac crest	NIL	24	76	NIL
2	Talectomy, Antibiotic Spacer Done At Different Hospital	NIL	NIL	Cap cephalexin six weeks	Complete talectomy	Trigen hind foot nail	Iliac crest	NIL	30	73	NIL
3	Partial Talectomy, Antibiotic Spacer	Yes	GNB (p. aeruginosa)	Iv piperacillin and tazobactam six weeks	Partial talus excision	Trigen hindfoot nail	Iliac crest	Wound infection (culture - p. aeruginosa)	24	66	Ankle debridement six months after arthrodesis Hind foot nail removal 10 months after arthrodesis
4	Partial Talectomy Antibiotic Spacer, Joint Spanning External Fixator	Yes	Coagulase negative staphylococcus	Cap cloxacillin six weeks	Partial talus excision	Trigen retrograde femur nail	Iliac crest	NIL	12	70	NIL

The patients were given a below-knee cast after suture removal for a period of 6 weeks after which they were mobilised with weight-bearing as tolerated to full weight bearing at 12 weeks.

## Results

The average follow-up period was 22.5 months (range: 12.0 – 30.0 months). The arthrodesis site had united in all these patients. No limb length discrepancy was detected except in Patient 3 where the operated limb was short by 3mm. Culture positive bacterial growth was present in Patients 3 and 4, who grew gram-negative bacteria (Pseudomonas aeruginosa) and coagulase-negative Staphylococci, respectively. Both these patients were administered culture appropriate antibiotics for a period of six weeks after the first stage of the reconstruction.

**Fig 1:**
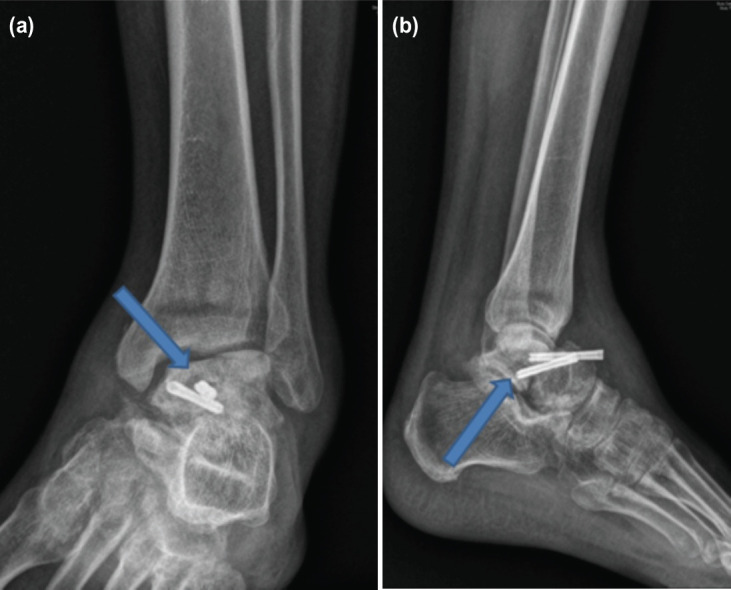
(a, b) Pre-operative AP and lateral radiographs of Patient 4.

**Fig 2:**
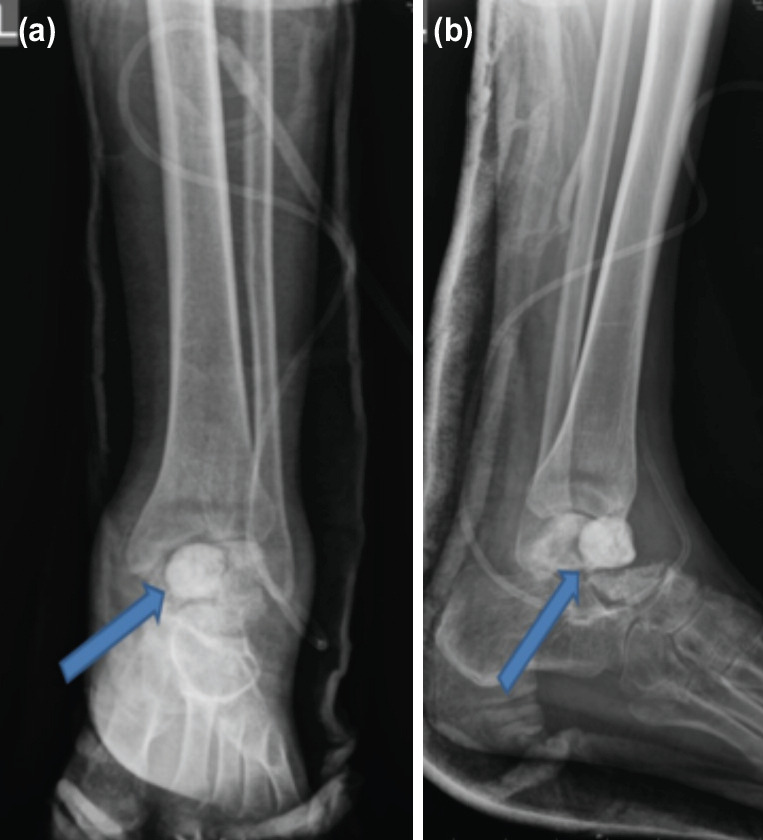
(a, b) 1st stage debridement and ALBC application with partial loss of talus in patient.

**Fig.3:**
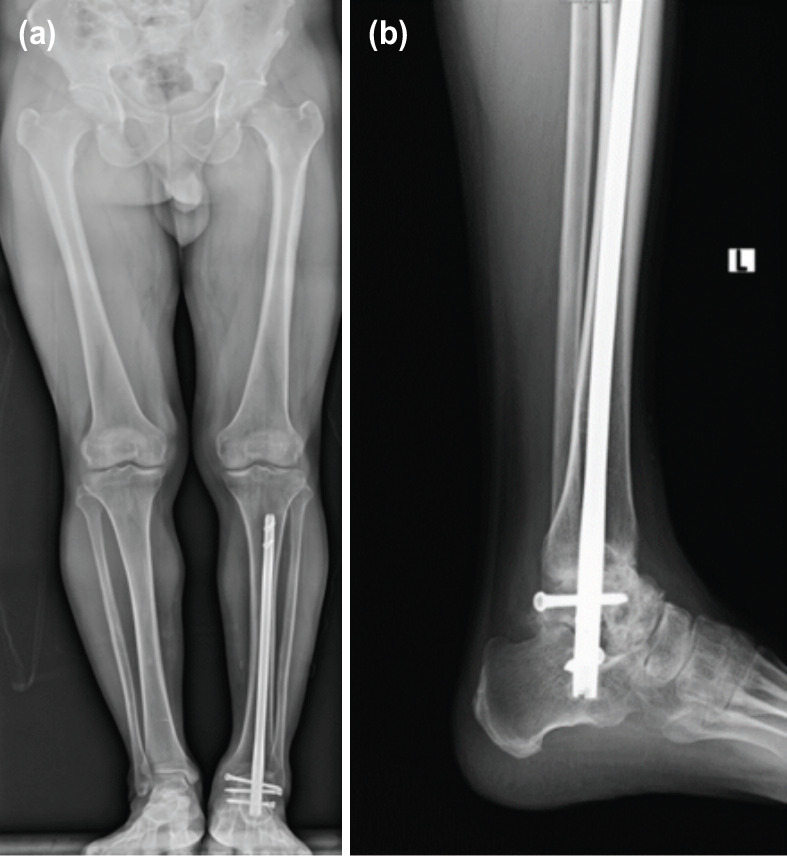
(a, b) AP and lateral radiographs of Patient 4 at follow-up of 12 months.

**Fig 4:**
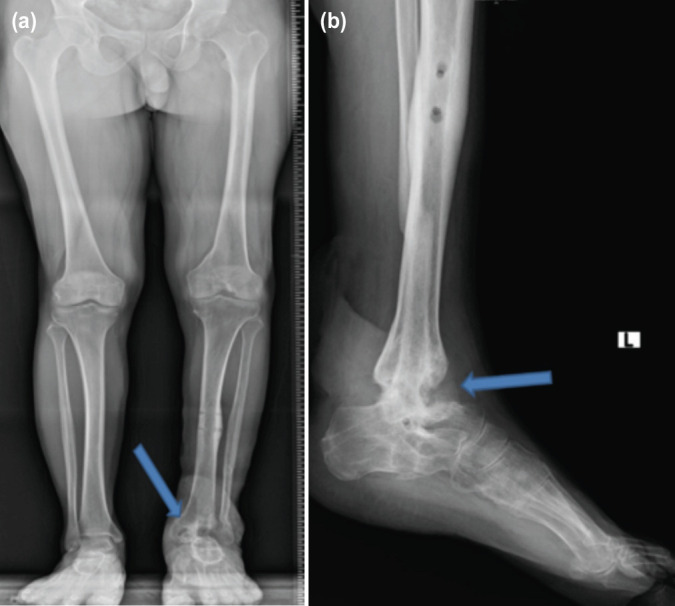
(a, b) Patient 3 after nail removal with arthrodesis site union at 24 months of follow-up.

**Fig 5:**
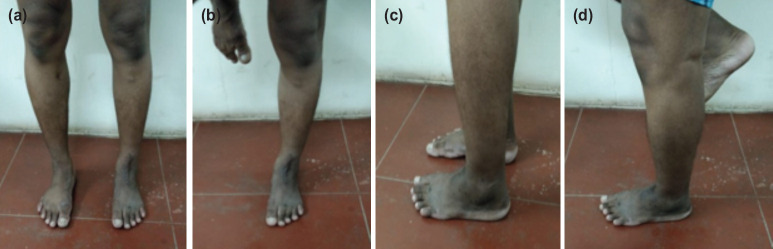
(a, b, c, d) Clinical pictures of Patient 4 at 12 months of follow-up.

Bony union was observed in all four patients between the tibial plafond and the talar remnant and calcaneum (Fig. 1 to 5).

The average AOFAS score was 71.25 (range: 70.0 – 76.0) which was acceptable in all the patients as shown in Table I. There was no limitation of knee movement in these four patients.

Patient 3 developed a wound infection of the ankle joint six months after the arthrodesis procedure. This patient underwent debridement of the ankle joint and was given culture appropriate antibiotics. He again developed an infection of the ankle joint 10 months after the arthrodesis procedure. The radiograph of the ankle indicated a bony union at the arthrodesis site. Clinical resolution of all the symptoms was achieved at this time after nail removal. He was advised full weight-bearing after removal of the implant (Fig. 4).

## Discussion

Salvage of post-traumatic or infective talus injuries with partial or complete loss is managed with debridement and tibio-calcaneal or tibiotalocalcaneal arthrodesis^10,17^. Kolker *et al* have described the staged management of three cases of talus osteomyelitis secondary to trauma using an external fixator and iliac crest bone grafting^28^. Jaffe *et al* have described four clinical scenarios of talar bone loss and osteomyelitis, all of whom were treated with four different techniques^29^. Klos *et al* have described the management of nine cases of talar bone loss with the hindfoot nail in a single stage^15^. Ochman *et al* have described the management of three patients with severe bone loss of the talus and the distal tibia with initial soft tissue cover and staged tibio-calcaneal arthrodesis with hind foot nails with an emphasis on the role of the intramedullary implant in the salvage of these limbs. Restoration of ankle height was not achieved in these patients as all of them underwent tibio-calcaneal arthrodesis through extramedullary and intramedullary implants with the remnant talus, if present, used as a bone graft^30^.

Preservation of ankle height in patients undergoing tibiotalocalcaneal (TTC) arthrodesis has been obtained by the use of the Ilizarov technique, ceramic/ metallic implants, femoral head allografts, 3D titanium cages, and 3D printed talus arthroplasty^10,20,22,23,31,32,33^. The advantages of maintaining the ankle height are that it restores the limb length, provides adequate tension to the soft tissues, and preserves the musculotendinous function of the foot^31^. In this series, we used trapezoidal or rectangular bone blocks from the patient’s iliac crest to maintain the height of the ankle joint. None of the patients had limb length discrepancy except Patient 3 who was short by 3mm. All the four patients had endured a minimum of two surgeries with three patients (Patients 2 and 3) undergoing reverse sural artery (RSA) flap for soft tissue coverage before presenting to us. Two of the patients (Patients 3 and 4) were found to have culture-positive growth after the first stage. We did not use the femoral head allografts to maintain the ankle height in these patients during the second stage of reconstruction to decrease the chance of residual infection. The 3D printed titanium cages and talus arthroplasty has an important role in the management of talus reconstruction and has shown to have good early outcomes^21-23^. These technological advances are not universally available, popular, and cause financial strain on the patient.

Rammelt *et al* has described the stages of post-traumatic reconstruction of talus non-union/malunion based on the presence of avascular necrosis (AVN) and infection^34^. Based on this classification, all four patients in this series fall under stage 5, where the authors have advised repeated debridement of the infected and necrotic bone resulting in complete or partial loss of the talus followed by fusion at a later stage. All the patients in our series except Patient 1 had multiple surgeries with ill-defined treatment history. We had maintained a high index of suspicion for infection even before initiating the first stage of debridement and ALBC spacer application. During the debridement, all of the necrotic and infected bone was removed resulting in partial remnant talus which showed good bleeding from the surface and still attached to the soft tissues. Hence, the arthrodesis was completed in a staged fashion.

The ankle joint was exposed by the anterolateral “universal” incision in all our patients. We did not use the transfibular approach as described by Klos *et al* or the posterior approach described by Abd-Ella *et al* since we wanted to avoid damage to the ankle syndesmosis and extensive dissection of the posterior structure^15,35^. Damage to the syndesmotic ligaments and the interosseous membrane increases the inversion and rotational stresses around the ankle joint resulting in deep muscular pain and weakness while walking^36^. Preservation of the fibula maintains the anatomy of the ankle joints and providing restraint to the peroneal tendons^37^.

All our patients were young males and involved in heavy manual work. We believe that maintaining the height of the ankle joint as well as avoiding injury to the syndesmotic ligaments during exposure helped in decreasing the morbidity of the involved limb. All the patients have acceptable AOFAS scores comparable to the literature available on the management of this rare condition, despite an infected nail removal in Patient 3 as shown in Table II. In patients 1 and 4, we used the retrograde femur nail for fixation instead of the dedicated hind foot nail to keep the surgical costs down. We found that the retrograde femur nails are a good alternative to the hind foot nails.

**Table II:** Comparison of AOFAS scores with other articles on the management of talus bone loss

Article	AOFAS score
Tibio- calcaneal arthrodesis with hind foot nail in severe loss of talus (Klos *et al*^15^)	71.5
Retrograde nail for tibiotalocalcaneal arthrodesis in severe bone loss of distal tibia and talus (Ochman *et al*^30^)	58.3
Ankle arthrodesis using Ilizarov method (Fragomen *et al*^10^)	71
Management of talus non-union with avascular necrosis with hind foot nail and bone grafting (Abd-Ella *et al*^35^)	76.6
Our paper	71.25

Staging the arthrodesis procedure, use of ALBC to maintain the ankle architecture during the first stage of debridement, use of culture appropriate antibiotics, autogenous dome filling bone blocks to maintain the height of the ankle, and an intramedullary nail device is a safe way to approach this difficult problem. A review of English literature did not reveal many studies where the talar bone loss or its complications were managed in two stages, using the bone blocks to maintain the height of the ankle and the nail device, although, each of these strategies has been applied individually by various authors. Abd-Ella *et al* has described the management of talus bone loss with hind foot nail and strut graft from the iliac crest in a single stage^35^. Clowers *et al* has described trapezoidal bone blocks fashioned out of femoral head allografts in the management of severe bone loss from the hind foot^38^. We hope that this study adds to the literature on the management of talar bone loss.

Limitations of this study are its retrospective nature, lack of comparative data, and the small number of patients. However, despite our appreciation of the limitations of our investigation, we believe that the results of this study could be useful in adding to the literature for the management of partial or complete talus bone loss secondary to the trauma associated with infection.

## Conclusion

In conclusion, staging the management of talus bone loss in patients with proven or even suspicion of infection is a well-heeled strategy. The use of autogenous rectangular or trapezoidal iliac crest bone blocks prevents the loss of ankle height.
